# Case report of a 72-year-old man with diaphragmatic hernia and thoracic gastropericardial fistula after esophagectomy for 18 years

**DOI:** 10.1186/s13019-021-01574-z

**Published:** 2021-07-07

**Authors:** Xinjian Xu, Zhaoyang Yan, Ming He

**Affiliations:** Department of thoracic surgery, The Fourth Hospital of Hebei Medical University, Shijiazhuang, 050011 China

**Keywords:** Esophagectomy, Diaphragmatic hernias, Thoracic gastropericardial fistula, Case report

## Abstract

**Background:**

Both diaphragmatic hernia and thoracic gastropericardial fistula rarely occur simultaneously in patients with radical esophagectomy.

**Case presentation:**

A 72-year-old man presented to our hospital with 1 day of nausea, vomiting and acute left chest pain. He had radical esophagectomy (Sweet approach) for esophageal cancer 18 years ago. Computed tomography (CT) of the chest revealed diaphragmatic hernias and air collection within the pericardial space. While an operation of diaphragmatic hernia repair was decisively performed to prevent further serious complications, unusually, a thoracic gastropericardial fistula was also found unusually.

**Conclusion:**

Diaphragmatic hernia and thoracic gastropericardial fistula may occasionally coexist in patients with esophagectomy. Upper GI radiograph with a water-soluble contrast agent is a better diagnostic tool than CT in visualizing the fistula.

## Background

Esophageal replacement with gastric conduit is a common surgical method of radical esophagectomy. Anastomotic leakage and conduit ischemia are mostly present in the early postoperative period of patients with esophagectomy. However, diaphragmatic hernias and conduit ulceration, as well as conduit fistulas are often observed separately at the emergency room as they have relatively longer survival times [1]. Both diaphragmatic hernia and thoracic gastropericardial fistula complications rarely occur simultaneously and both diagnoses could be missed, especially in an emergency setting.

## Case presentation

**Fig. 1:**
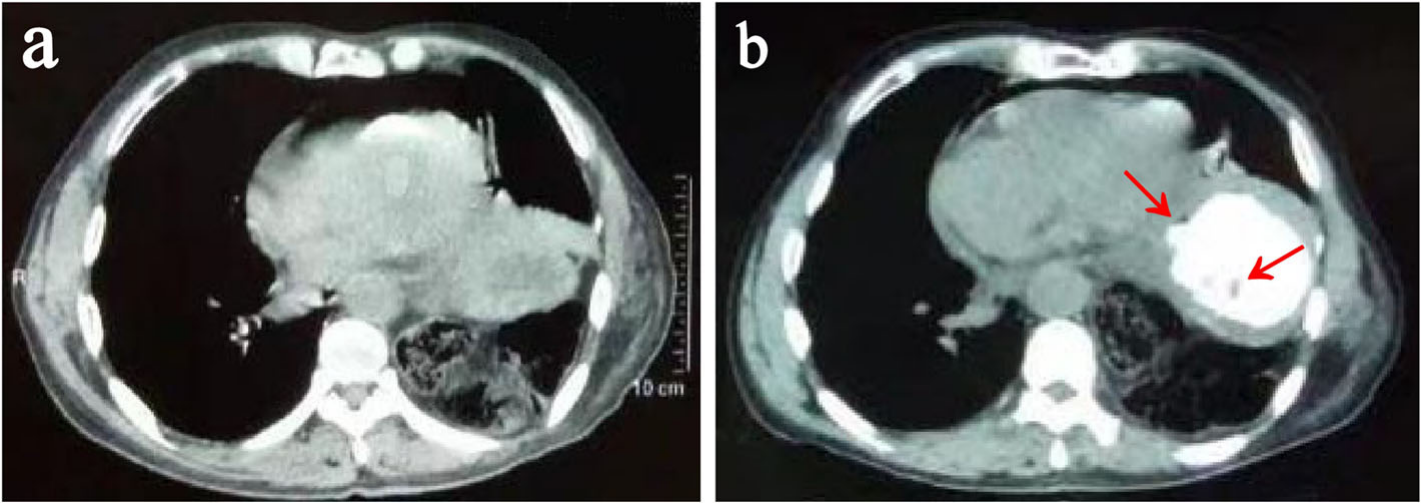
Preoperative CT. **a** CT revealed the diaphragmatic hernias and air collection within the pericardial space. **b** There was no contrast agent leak into the pericardial space while a gastric wall ulcer and an unspecific low density in the thoracic gastric cavity were noticed

**Fig. 2:**
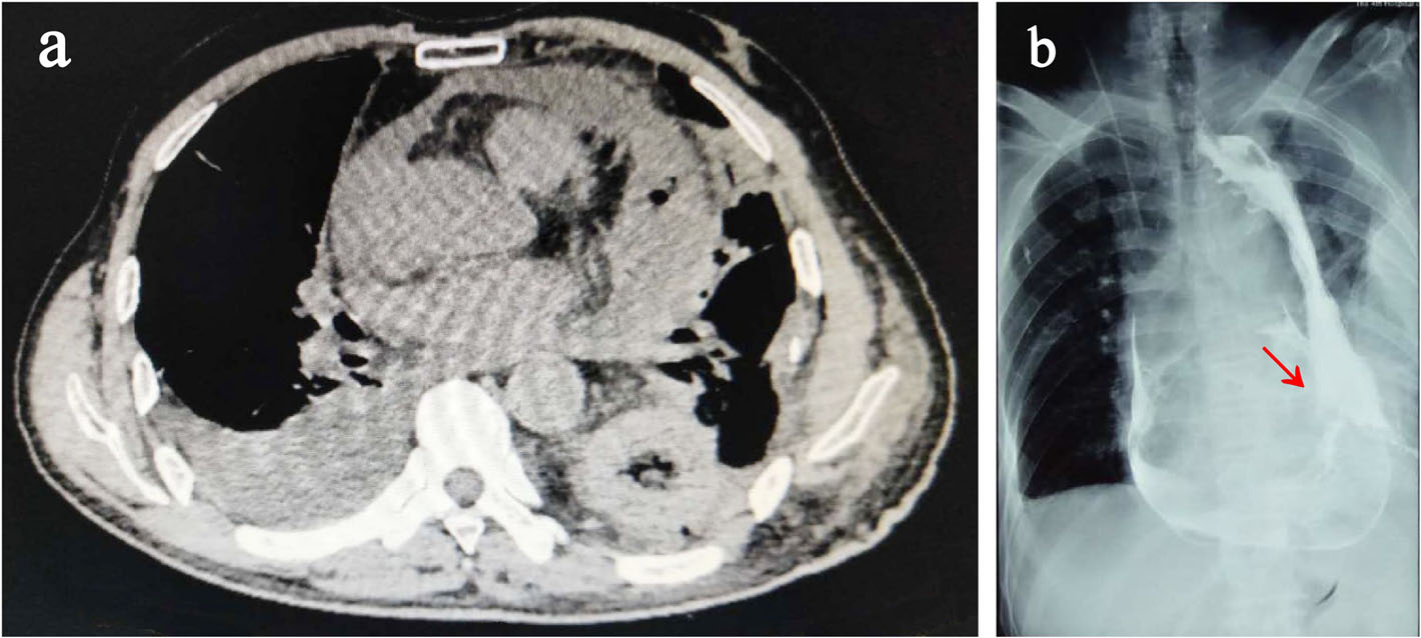
CT and contrast radiography revealed a thoracic gastropericardial fistula. **a** CT showed moderate pericardial effusion and right pleural effusion. **b** Contrast roentgenogram showed a thoracic gastropericardial fistula

**Fig. 3:**
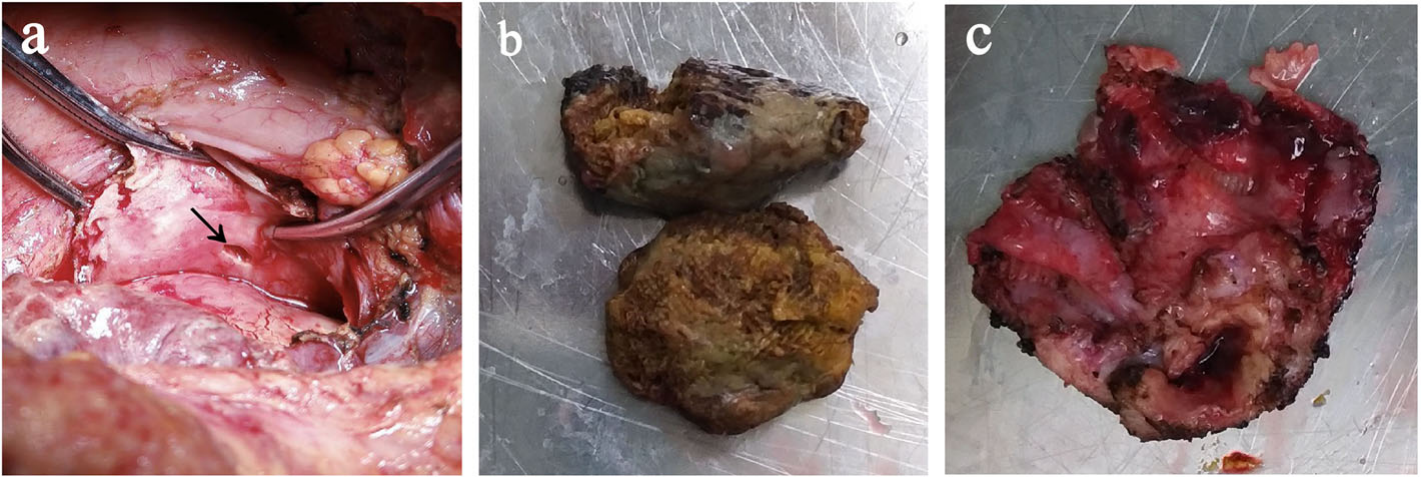
Thoracic gastropericardial fistula and bezoars during operation. **a** Fistula tract during surgery. **b** Bezoars in the gastric tract. **c** Part of pericardium and gastric wall

A 72-year-old man, who had radical esophagectomy (Sweet approach) for esophageal cancer 18 years ago, was presented to the emergency department with 1 day of nausea, vomiting and acute left chest pain. Computed tomography (CT) revealed a diaphragmatic hernia and air collection within the pericardial space (Fig. 1a). Oral meglumine amidotrizoate was taken by the patient; however, the following CT did not reveal any contrast agent leak into the pericardial space, while a gastric wall ulcer and an unspecific low density in the thoracic gastric cavity were noticed (Fig. 1b). At the emergence room, his heart rate was 110, BP was 93/68 mmHg and labs showed HGB 101.8 g/L, WBC 11.1 × 10^9^/L, NE 10.18 × 10^9^/L, NE% 91.2, RBC 3.97 × 10^12^/L. Based on these results, a diaphragmatic hernia could be diagnosed, but the etiology of the presenting pneumopericardium was unknown. Given that the diaphragmatic hernias needed urgent surgery and that they were possibly related to the pneumopericardium, we repaired them with a right transthoracic approach and found that there was no strangulation of the intestines. The patient recovered quickly after surgery until, on the third postoperative day, he complained of new chest pain. New CT was ordered and showed moderate pericardial effusion and right pleural effusion, which led us to suspect the existence of a thoracic gastropericardial fistula (Fig. 2a). The following contrast roentgenogram confirmed the thoracic gastropericardial fistula (Fig. 2b). A 1 cm diameter fistula tract was observed in the resulting pericardium surgery through the left thoracotomy (Fig. 3a). In the surgery, we removed a nearly 5 cm diameter pericardium around the fistula and part of the gastric wall, and we found two bezoars in the gastric tract (Fig. 3b-c). After we took out the bezoars and placed a jejunal feeding tube, we repaired the gastric wall and covered it with mediastinal fat tissue. Finally, a fine tube was placed in the pericardium for the purpose of postoperative rinsing and a drainage tube was also placed at the pericardial defect region. With the daily pericardial flush, the patient recovered quickly after starting to take oral food on the seventh postoperative day.

## Discussion and conclusions

Esophageal replacement with gastric conduit is a common surgical methodof radical esophagectomy. In these patients, anastomotic leakage or conduit ischemia often develops in the early postoperative period, while diaphragmatic hernias, anastomotic stricture, conduit ulceration, and dysfunctional conduits frequently seen as the late complications. Among these, the gastric conduit ulceration could progress to a life-threatening gastropericardial fistula and diaphragmatic hernias, which clearly require urgent surgical care [2].

Gastropericardial fistulas are a rare and severe complication of radical esophagectomy with a mortality rate greater than 50% [3]. Clinical presentation of gastropericardial fistulas is often non-specific and includes dyspnea, chest pain and sudden death [4]. Our patient complained of left chest pain, nausea and vomiting, which are also the clinical manifestations of diaphragmatic hernias. Therefore, the diagnosis of a gastropericardial fistula could be missed while it coexists with diaphragmatic hernias, especially in an emergency setting.

Imaging and endoscopy can be used to assist the diagnosis of gastropericardial fistula. CT imaging often shows the cardiac enlargement and air or air-fluid levels in the pericardial cavity, while radiography with water soluble contrast can allow the fistula tract to be visualized. Also, gastroscopy can accurately reveal the ulceration or fistula tract [5]. In this case, the gastroscopy could have provided greater assistance with the diagnosis, although it was understandably very difficult to have in an emergency setting.

Surgical treatment is usually considered as an effective way to quickly improve the prognosis of a gastropericardial fistula [6]. In this case, we had resected a part of the pericardium and repaired the gastric wall and the patient recovered rapidly.

In conclusion, diaphragmatic hernias and thoracic gastropericardial fistulas could be present simultaneously in patients with a relatively long history of radical esophagectomy. CT in combination with radiography with a water-soluble contrast can enable a more rapid and accurate diagnosis for these patients.

## Data Availability

Not applicable.

## Abbreviations

CT: Computed tomography
BP: Blood pressure
WBC: White cell count
NE: Neutrophil count
NE%: Neutrophil ratio
RBC: Red cell count
HGB: Hemoglobin
